# Prescription stimulants for Medication-assisted treatment of Cocaine Use Disorder: A Case study and review of literature

**DOI:** 10.1192/j.eurpsy.2026.10489

**Published:** 2026-07-31

**Authors:** L. Li

**Affiliations:** Psychiatry and Behavioral Neurobiology, University of Alabama at Birmingham, Birmingham, United States

## Abstract

**Introduction:**

Cocaine use disorder (CUD) remains a challenging condition to treat, with high rates of relapse despite various interventions and without any FDA-approved treatments. Psychostimulants, such as dextroamphetamine, have emerged as a promising pharmacological treatment to reduce relapse and cravings in CUD.

**Objectives:**

We will investigate potential treatment options for CUD and highlight a patient case study as well as a review of literature.

**Methods:**

The Institutional review board approval was waivered. Electronic Medical Records were reviewed for this case. Our case study focuses on a 43-year-old male who was diagnosed with ADHD in childhood. He had been prescribed dextroamphetamine for most of his adolescent and adult life, until he began using cocaine two years ago. He reported using one to two grams of cocaine daily via insufflation. After this initial use, the patient was unable to remain in remission from cocaine use for more than two weeks at a time. The patient also had a history of occasional cannabis use. His use of cocaine caused significant damage to his social, emotional, and occupational functioning. Given the patient’s history of tolerating and responding well to dextroamphetamine-amphetamine salts for ADHD, it was decided that he would restart this medication for both ADHD and as a potential treatment for CUD.

**Results:**

At the initial visit, Dextroamphetamine-amphetamine salts were started at 10 mg twice daily and titrated to 20 mg three times daily (instant release formulation) over the course of 4 months. Following initiation, the patient was required to repeat urine drug screens (UDS) monthly at each clinical visit. UDS was positive for cocaine prior the initiation but remained negative on all subsequent tests. At each visit, the patient completed the Cocaine Craving Scale (CCS), a validated self-report questionnaire used to assess current craving intensity. The initial CCS score was 45, the highest possible score. His CCS scores decreased to 3-21 over the subsequent 6 months, and has since helped the patient maintain sobriety.

**Conclusions:**

Currently there are no FDA approved treatments for CUD. Dextroamphetamine shows promise as a potential pharmacological option for preventing relapses and cravings in certain subsets of individuals with CUD. Clinicians must weigh the risks of stimulant misuse with the potential therapeutic benefits in the context of a comprehensive treatment approach.

**Disclosure of Interest:**

None Declared

